# From Flow to Function: Using Different Static Mixers to Fabricate Microarchitected Materials Via Chaotic Printing

**DOI:** 10.1002/advs.76626

**Published:** 2026-07-29

**Authors:** Edna Johana Bolívar‐Monsalve, Carlos Fernando Ceballos‐González, Diego Alonso Quevedo‐Moreno, Irving Isaí Rendón‐Moreno, Ariel Cantoral‐Sánchez, Héctor Ambriz‐González, Mario Moisés Alvarez, Grissel Trujillo‐de Santiago

**Affiliations:** ^1^ Centro de Biotecnología‐FEMSA Tecnólogico de Monterrey Monterrey Nuevo León México; ^2^ Departamento de Mecatrónica Escuela de Ingeniería y Ciencias Tecnológico de Monterrey Monterrey Nuevo León México; ^3^ Expedition‐FEMSA Advanced Biofabrication Tecnológico de Monterrey Monterrey Nuevo León México; ^4^ Departamento de Bioingeniería Escuela de Ingeniería y Ciencias Tecnológico de Monterrey Monterrey Nuevo León México

**Keywords:** chaos, kenics, microarchitecture, printing, Ross, SMX, static mixer

## Abstract

Nature builds functional materials through simple yet powerful processes that generate structured architecture across scales—from the lamellar patterns in seashells to the zonal organization of living tissues. Emulating such complexity in engineered systems remains challenging and often requires microfabricated components, external fields, or specialized hardware. Previously, we introduced chaotic printing as a deterministic and flow‐ and geometry‐driven strategy for fabricating structured filaments, using static mixers embedded within extrusion printheads—primarily in the context of biofabrication. We broaden the architectural and functional scope of chaotic printing by exploring diverse static mixer designs and demonstrating its compatibility with three distinct deposition modes: wet‐printing, dripping, and direct ink writing. These modalities enable the generation of material constructs with chemically and biologically relevant internal organization. We showcase examples ranging from zonally arranged mammalian cells that prefigure microtissue compartments to spatially patterned bacterial consortia composed of strict and facultative anaerobes and localized mineral precipitation within hydrogel filaments. These proof‐of‐concept‐demonstrations underscore the potential of chaotic printing for fabricating structured soft matter where internal microarchitecture enables biologically and chemically relevant processes. This study positions chaotic printing as a modular, scalable, accessible platform for generating architected materials across fields ranging from cell culture and microbiology to functional soft materials.

## Graphical abstract

Complex microarchitectures emerge from simple flow‐driven transformations as static mixers iteratively reorient and split material domains. Coupled with diverse deposition strategies, chaotic printing provides an accessible platform for fabricating architected materials that support biologically and chemically relevant processes.

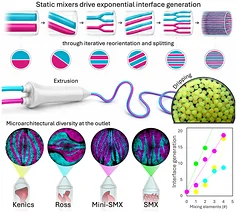

## 1 Introduction

Nature offers a powerful framework for building complex and functional structures. From the hierarchical arrangement of collagen and minerals in bone to the layered architectures of plant cuticles and the mineralized ultrastructure of seashells, natural systems achieve remarkable performance by organizing matter through gradients, interfaces, and zonal compartmentalization. These sophisticated yet elegant architectures often emerge from simple, iterative, and self‐organizing processes.

These principles are not exclusive to biological tissues. Comparable patterns of structure formation are found in geophysical systems like Earth's mantle and even in cosmic phenomena such as spiral galaxies or Turing‐like morphogenetic structures in astrophysical plasmas [1, 2, 3].

In contrast, engineered systems often require intricate and energy‐intensive technologies to replicate similar levels of internal complexity. While these methods can deliver high precision, their implementation is frequently constrained by cost, scalability, and accessibility [4, 5, 6]. How might we reproduce nature‐like structural richness using fabrication approaches that are more accessible and scalable?

Many areas of science and engineering increasingly demand materials with precisely defined internal architecture. In tissue engineering, zonal microenvironments, vascular networks, and structured interfaces between cell types are critical to function. In microbial systems, the spatial arrangement of consortia—as seen in biofilms, soil, or gut ecosystems—can elicit spatially induced behaviors. Similarly, in catalysis and soft matter systems, localizing enzymes, ions, or other reactive species can enhance selectivity and efficiency, as demonstrated by recent strategies involving ionogels with compartmentalized functionalities [3, 7, 8, 9].

Recent studies clearly demonstrate that internal architecture is a powerful design variable in functional composites [10, 11, 12]. These contributions represent elegant examples of structure‐guided functional materials, particularly in the topic of thermally conductive and electromagnetic shielding composites.

Several advanced fabrication strategies have been developed to create internal microarchitecture. Two‐photon polymerization offers nanometric resolution but remains expensive and low‐throughput [13]. Digital micromirror device‐based photopatterning provides spatial precision yet requires complex optical systems and specialized resins [14]. Multiphase microfluidics enables dynamic interface control, though it often depends on custom‐built setups [15]. Field‐based patterning methods—including electric, magnetic, and acoustic techniques—can yield finely structured architectures, but their applicability is limited by material compatibility and equipment requirements [16].

While these advanced methods have pushed the boundaries of resolution and spatial control, they are often constrained by cost, throughput, and technical complexity. Surprisingly, few approaches have sought to achieve comparable structural sophistication using simpler and more accessible means—an opportunity that remains largely untapped.

In this context, our group has developed continuous chaotic printing, a strategy that uses mixers capable of inducing chaotic flows, including static mixers integrated into extrusion printheads. As materials flow through these mixers, they are passively split and reoriented under laminar flow conditions, producing fine and reproducible internal layers [17, 18, 19].

Each mixing element duplicates existing material interfaces, leading to exponential growth in the number of layers across the length of the mixer. Unlike other methods that require microstructured nozzles or external fields, chaotic printing is simple, scalable, and mathematically modelable. It is compatible with a wide range of materials, including polymers, particle‐laden hydrogels, and even bioinks containing fragile mammalian cells [20, 21, 22].

In this work, we build on this platform to explore and expand the architectural diversity achievable through chaotic printing. We introduce additional types of static mixers—originally developed for industrial mixing—that had not been previously used in biofabrication or in the fabrication of architected soft materials. By incorporating these mixers into printheads, we demonstrate the generation of a broader library of internal patterns. We also validate the adaptability of chaotic printing across three distinct deposition modes: continuous wet‐printing, where filaments are extruded and crosslinked in a calcium bath; dripping, where discrete spherical constructs form by droplet detachment; and direct ink writing, where viscoelastic materials are patterned using pressure‐driven extrusion [23, 24, 25].

These platforms are used to create constructs with functional spatial organization. In one set of experiments, mammalian cells are printed in structured arrangements that prefigure microtissue zonation. In another, bacteria are compartmentalized by positioning strict anaerobes in the central region and facultative anaerobes toward the periphery, providing a proof‐of‐concept for the rational design of synthetic microbial consortia. Finally, we demonstrate localized mineral precipitation inside hydrogel fibers, a process with potential applications in biomineralization, catalysis, and the fabrication of bioinspired composite materials.

Altogether, this work positions chaotic printing as a flexible and accessible strategy for producing complex internal architectures through passive, scalable mechanisms. We anticipate that this approach will be of broad interest to researchers in bioprinting, soft materials, catalysis, additive manufacturing, and synthetic biology—and we hope it inspires further exploration of chaotic flows as tools for advanced material design.

## 2 Results and Discussion

### 2.1 Chaotic Printing Using Different Static Mixers

To demonstrate the versatility of continuous chaotic printing beyond the use of traditional Kenics static mixers, we used printheads equipped with four different static mixer geometries—Kenics, Ross, Mini‐SMX, and SMX (Figure 1A)—across three representative fabrication modes: fiber extrusion, dripping, and direct writing (Figure 1B) [26, 27, 28, 29].

**FIGURE 1:**
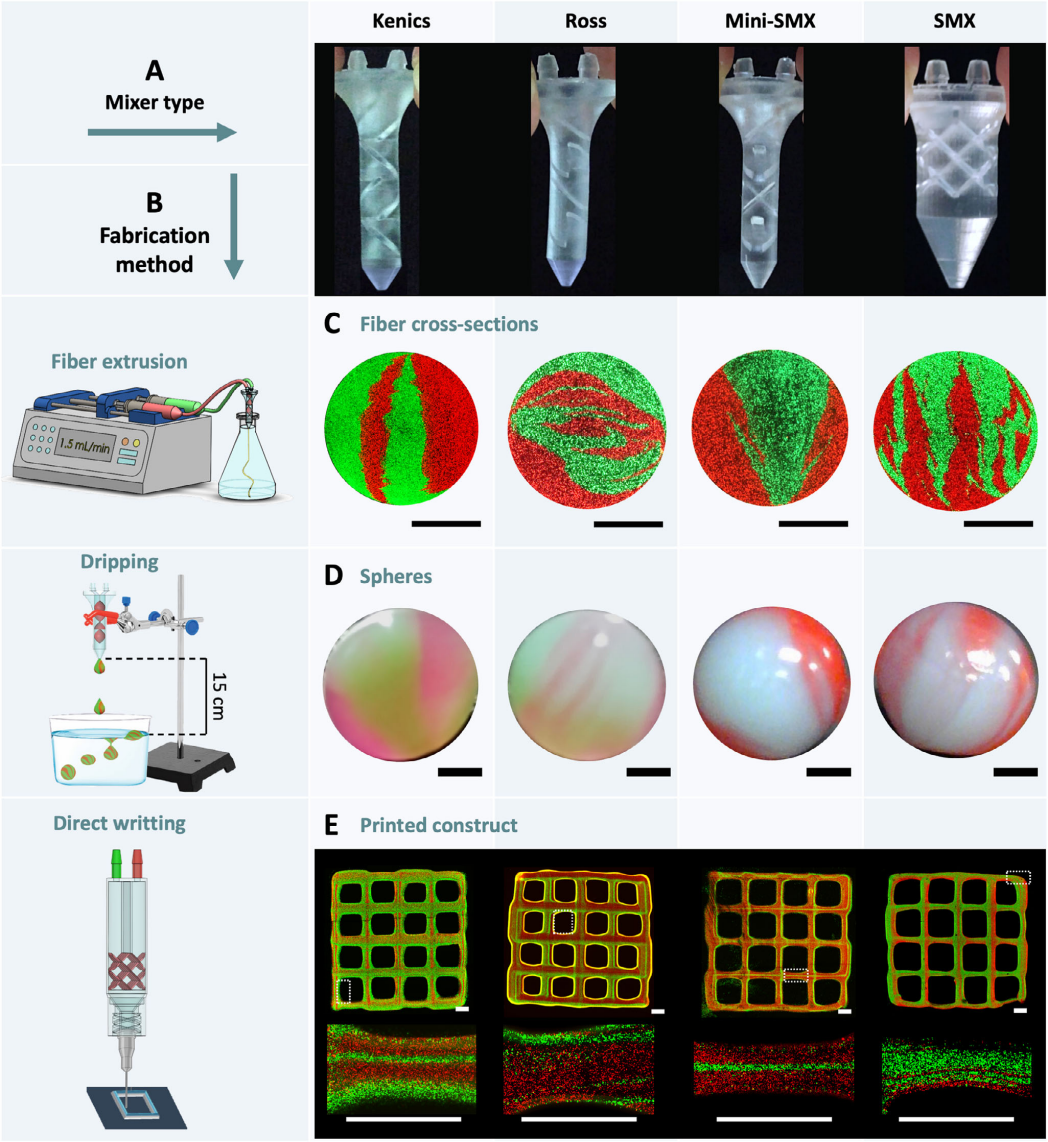
Demonstration of the versatility of continuous chaotic printing using different static mixers and fabrication modalities. (A) Static mixer types used in this study: Kenics static mixer, Ross mixer, Mini‐SMX, and SMX. (B) Fabrication modalities evaluated: fiber extrusion, dripping, and direct writing. (C) Cross‐sectional images of hydrogel fibers extruded using each mixer type (scale bar: 500 µm). (D) Microstructured spherical gels obtained by dripping with each mixer type (scale bar: 1000 µm). (E) Top‐view and longitudinal section of 4 × 4 hydrogel grids printed by direct writing with each mixer type. All constructs were printed using a 2% (w/v) sodium alginate solution (scale bar: 1000 µm).

All mixers tested enabled the fabrication of hydrogel constructs with preserved internal microstructures, underscoring the adaptability of the printing system to different physical configurations.

Cross‐sectional images of extruded fibers (Figure 1C) revealed distinct internal striation patterns for each mixer, showing that flow architecture can be modulated through mixer design. In the dripping setup, spherical hydrogels formed upon contact with the crosslinking bath retained their internal architecture (Figure 1D), even though the formation process might be expected to disrupt microstructural integrity. This structural preservation, consistent with previous observations [24], confirms the robustness of chaotic interfaces during dripping‐based fabrication.

In the direct writing modality, all mixers supported the formation of grid‐like constructs composed of hydrogel filaments, each maintaining discernible internal microstructure (Figure 1E). These results show that the preservation of fine inner features can be achieved even during more complex deposition strategies such as extrusive 3D (bio)printing.

Together, these findings highlight the modularity and functional design potential of continuous chaotic printing using diverse static mixer types. This flexibility broadens the range of achievable geometries and supports application‐driven customization based on required resolution, material properties, and fabrication constraints.

To systematically explore how mixer geometry and the number of mixing elements influence internal patterning in chaotic printing, we characterized printheads containing four static mixer types—Kenics, Ross, Mini‐SMX, and SMX—using configurations with increasing numbers of internal elements. For each mixer, we describe the shape of the individual mixing elements and their spatial arrangement within the assembled mixer head (Figure 2A–Di, ii). These geometries vary in curvature, angularity, and overall complexity, which can influence flow trajectories and the resulting internal structures (Figure 2iii).

**FIGURE 2:**
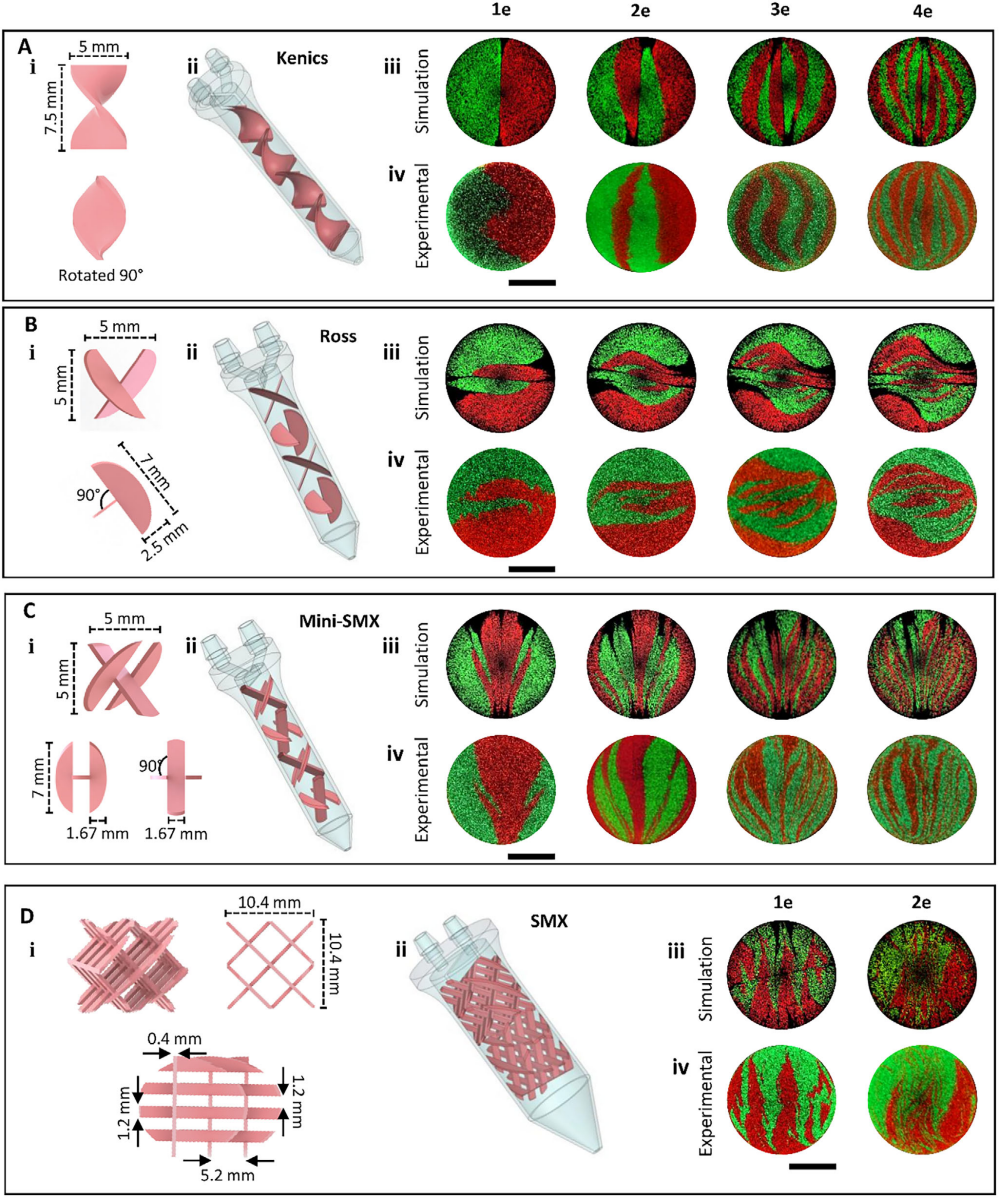
Effect of mixing element design and number on internal structure in chaotic printing. Analysis of four different static mixers: (A) Kenics, (B) Ross, (C) Mini‐SMX, and (D) SMX. For each mixer: (i) Morphology of the individual mixing element from different perspectives, highlighting its geometry. (ii) Assembly view of the static mixer head containing the corresponding elements. (iii) Computationally simulated cross‐sections showing the internal structure at the mixer outlet after 1 to 4 mixing elements (only 1 and 2 for SMX). (iv) Experimental cross‐sections of hydrogel fibers printed with the same mixer configurations, using fluorescently labeled inks. (Scale bar: 500 µm).

Experimentally, increasing the number of mixing elements led to progressively finer and more intricate internal architectures across all mixer types (Figure 2iv). In the case of the Kenics mixer, this refinement resulted in aligned, multilayered patterns reaching feature sizes of ∼60 µm, with four elements yielding 16 layers within ∼1 mm. Other mixer geometries enabled even finer structuring; for example, the Mini‐SMX achieved characteristic feature sizes down to ∼10 µm when using 3–4 elements in specific configurations.

These geometries offer new opportunities for creating bioinspired materials in which architecture directly contributes to function [30, 31, 32, 33, 34, 35].

Each static mixer generates a distinctive microarchitectural fingerprint. The Kenics static mixer produces a multilayer microstructure that has been widely described in previous work (Figure 2A) [20, 23, 36, 37]. In contrast, the Ross, Mini‐SMX, and SMX mixers (Figure 2B–D) yield visually distinct patterns with more organic characteristics. Ross‐based constructs exhibit asymmetric, wave‐ or flame‐like architectures (Figure 2B), while the Mini‐SMX generates symmetric motifs reminiscent of flowers or radiant forms (Figure 2C). The SMX mixer, evaluated with one and two mixing elements, produces flame‐like structures, with pronounced segregation of ink phases toward opposite sides of the cross‐section observed only in the two‐element configuration (Figure 2D).

Computational Fluid Dynamics (CFD) simulations of the cross‐sectional patterns at the outlet of each mixer (Figure 2iii) closely mirrored the experimental cross‐sections of printed hydrogel fibers (Figure 2iv), confirming the reproducibility and predictive power of the computational modeling. This agreement emphasizes the deterministic behavior of chaotic advection, even when using geometrically distinct mixer designs. The simulations presented in Figure 2 were not intended to represent stochastic or ensemble‐averaged behaviors, but rather representative steady‐state solutions illustrating the characteristic flow‐induced spatial organization generated by each mixer geometry. The computational framework assumes incompressible laminar flow using fluid properties comparable to those of the pre‐gel solutions employed experimentally, whose viscosities are close to that of water under the printing conditions used in this work. Under these conditions, the resulting microarchitectures are primarily dictated by the geometry and topology of the static mixer rather than by random perturbations or transient fluctuations. This deterministic behavior is, in fact, one of the defining characteristics of chaotic advection under laminar flow conditions, where complex spatial patterns emerge from repeated stretching and folding or reorientation and splitting of fluid streams despite the absence of turbulence or moving mechanical components. Consistent with this interpretation, the experimentally observed cross‐sectional architectures closely matched the computationally predicted patterns across the different mixer geometries evaluated, supporting the reproducibility and predictive value of the simulations despite inevitable minor experimental variability.

Operational parameters such as viscosity, temperature, and flow rate can influence the fidelity and stability of the experimentally generated structures, although the overall architectural motifs remain strongly governed by the mixer geometry itself.

Beyond its structural versatility, this level of resolution and internal complexity would be difficult to achieve using conventional extrusion‐based 3D printing. Reproducing similar architectures would require ultra‐fine nozzles and sequential, layer‐by‐layer deposition of distinct domains—such as the green and red inks—which is impractical for continuous filament fabrication and would involve significant time and technical constraints [38, 39]. In contrast, chaotic printing enables the simultaneous, high‐throughput deposition of interleaved materials in a single step. Moreover, as demonstrated in Figure 1, chaos‐based fabrication can be integrated into a wide range of manufacturing modalities, including wet spinning, dripping, direct extrusion, and potentially even microfluidic or light‐based systems [19, 24].

### 2.2 Flow Characterization

In the context of chaotic printing, the primary goal is not merely to achieve homogenization in the classical mixing sense but to rapidly generate extensive interfacial area and intricate ordered microarchitecture within hydrogel filaments. Static mixer geometry plays a decisive role in this process by dictating how inlet streams are reoriented and divided along the flow path. These deformation cycles, characteristic of chaotic advection, exponentially multiply the interface (contact) surfaces between materials, enabling the formation of highly structured constructs over short axial distances.

Next, to assess the potential of different mixer designs for this purpose, we examined their velocity fields, wall shear stress distributions, associated pressure drops, and ability to generate microarchitecture per mixing unit (per static mixer). This integrated analysis will establish that different static mixers exhibit different balances between interface proliferation and energetic cost—two parameters that are critical for scaling chaotic printing toward advanced manufacturing applications.

Figure 3 shows a computational fluid dynamics (CFD) analysis of the velocity (Figure 3A), wall shear stress (Figure 3B), and pressure drop (Figure 3C) fields generated by each one of the static mixer types used in our experiments.

**FIGURE 3:**
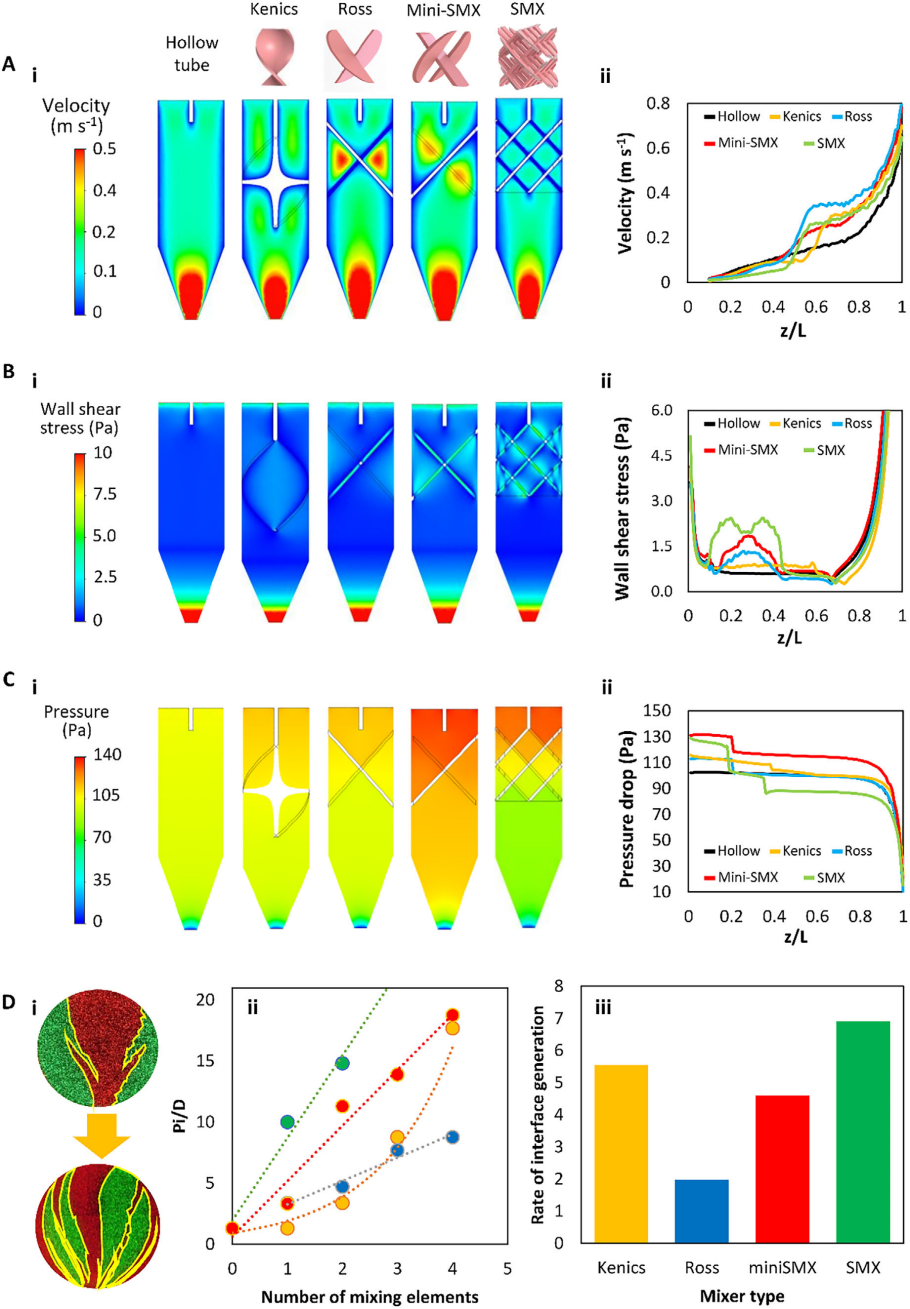
Fluid dynamics and interface generation in chaotic printheads containing different static mixers. (A) Velocity fields obtained from CFD simulations for printheads containing a single unit of different static mixing elements: hollow tube (included for reference), Kenics, Ross, Mini‐SMX, and SMX. (i) Longitudinal sections showing the internal velocity magnitude (m s^−^^1^). (ii) Velocity magnitude along the axial coordinate (z), normalized by the total printhead length (z/L). (B) CFD‐calculated wall shear stress distribution. (i) Longitudinal sections showing wall shear stress (Pa). (ii) Wall shear stress profiles along the normalized axial coordinate (z/L). (C) Pressure distribution within the printheads. (i) Longitudinal sections showing internal pressure (Pa). (ii) Pressure drop profiles along the normalized axial coordinate (z/L). (D) Quantification of interface generation between two co‐extruded materials. (i) Schematic representation of the intermaterial perimeter (Pi), defined as the internal boundary separating the red and green domains. (ii) Evolution of the intermaterial perimeter (Pi), as estimated through image analysis, as a function of the number of mixing elements in the printhead, calculated by image analysis of experimental cross‐sectional micrographs obtained from filaments generated using printheads equipped with different static mixers. (iii) Short‐term linear rates of interface generation derived from the slopes of the curves shown in panel D(ii).

### 2.3 Analysis of Velocity Fields and Wall Shear Rates

The computed velocity fields for single‐element configurations of each static mixer geometry reveal distinct flow organizations within the printheads (Figure 3A–Ci). In the reference case of the hollow tube, the velocity field remains nearly uniform across the central plane, with negligible transverse variations. This behavior is characteristic of laminar pipe flow and indicates minimal stretching or deformation of the inlet streams [40, 41]. The incorporation of static mixing elements significantly alters the velocity distribution. In the Kenics mixer [37], the helical geometry induces continuous rotational reorientation of the flow, redistributing velocity across the cross‐section and producing a characteristic swirling pattern and results in relatively smooth velocity gradients [42, 43]. In contrast, the Ross mixer generates sharper velocity transitions due to abrupt directional changes imposed by the mixing blades. These features create localized zones of accelerated flow adjacent to blade edges, producing strong velocity gradients that promote high interface stretching [29, 44]. The SMX and Mini‐SMX mixers generate even more complex velocity fields [45, 46]. Their intersecting baffle structures drive strong transverse flows and repeated flow reorientation, producing multiple regions where streamlines converge and diverge—conditions favorable for rapid interfacial proliferation. We also determined the velocity magnitude along the central *z*‐axis of each mixer and plotted these values against the normalized mixer length (*z/L*) (Figure 3Aii).

Overall, this analysis also suggests that the Ross mixer is the static mixer that induces the highest variations in velocity magnitudes, while the Kenics, mini‐SMX, and SMX mixers induce less aggressive velocity gradients. Sudden changes in speed can compromise structural or biological integrity and therefore are particularly relevant when handling non‐Newtonian fluids or sensitive systems such as mammalian cells [47, 48].

Wall shear rate maps (Figure 3Bi) identify regions where strong viscous stresses are imposed on the fluid as it interacts with the solid boundaries of the mixer elements. These stresses are relevant for evaluating mechanical loading and potential cell damage in bioprinting applications [47, 48].

The axial profiles of wall shear rate are presented in Figure 3Bii. The Kenics design produces a smoother shear distribution, whereas the Ross and SMX configurations generate sharper peaks corresponding to the locations where flow is strongly redirected by mixer elements.

### 2.4 Pressure Drop and Energetic Cost

Pressure drop profiles (Figure 3Ci) provide a measure of the energetic cost associated with each mixer geometry [29, 49]. The hollow channel exhibits a smooth pressure gradient typical of fully developed laminar pipe flow, with a final increase near the nozzle exit caused by the abrupt reduction in diameter. The Kenics and Ross mixers introduce additional pressure losses associated with flow redirection around their internal elements, but their overall pressure drops remain comparable and only moderately larger than that of the hollow channel. In contrast, the SMX and Mini‐SMX geometries produce significantly larger pressure drops due to the repeated obstruction and transverse redirection of the flow by their intersecting baffles.

The integrated pressure drop curves (Figure 3Cii) therefore highlight the trade‐off between interface generation and energetic cost. While SMX‐type mixers generate strong velocity gradients and complex flow structures favorable for interface proliferation, they do so at the expense of substantially higher‐pressure losses [49]. The Kenics geometry, by contrast, achieves effective flow reorientation with a comparatively moderate pressure penalty, making it attractive for applications where microarchitecture generation and energetic efficiency must be balanced [49].

Collectively, these results demonstrate that static mixer geometry simultaneously controls velocity gradients, mechanical stresses, and pressure losses within chaotic printheads [29, 49], revealing a fundamental trade‐off between interface generation, energetic efficiency, and mechanical loading that must be carefully balanced when designing systems for microarchitectural biofabrication. Moreover, differences in velocity organization, shear distribution, and pressure losses ultimately influence the rate at which new interfaces are created within the printed filaments [49]. To evaluate this effect quantitatively, we next examine the interface generation rates associated with each static mixer geometry.

### 2.5 Comparative Performance in Interface Generation

In the context of chaotic printing, the amount of interfacial area generated within short residence lengths directly determines the attainable resolution of internal microarchitecture.

The analysis presented in Figure 4 reveals marked differences in the ability of each static mixer geometry to generate fine‐scale interfaces and intricate microarchitecture, which constitutes the primary functional objective in chaotic printing. We quantified the interface as the total perimeter separating red and green domains in the cross‐sectional images shown in Figure 2, (illustrated by the yellow contour in Figure 3Di). This interfacial length was calculated for hydrogel filaments fabricated using printheads equipped with the different static mixer geometries and containing different numbers of mixing elements (Figure 2A–D). The analysis was intentionally restricted to a relatively small number of elements, as this operational regime is the most relevant for biofabrication applications where the goal is to generate microarchitectures with characteristic dimensions in the tens‐to‐hundreds of micrometers range.

**FIGURE 4:**
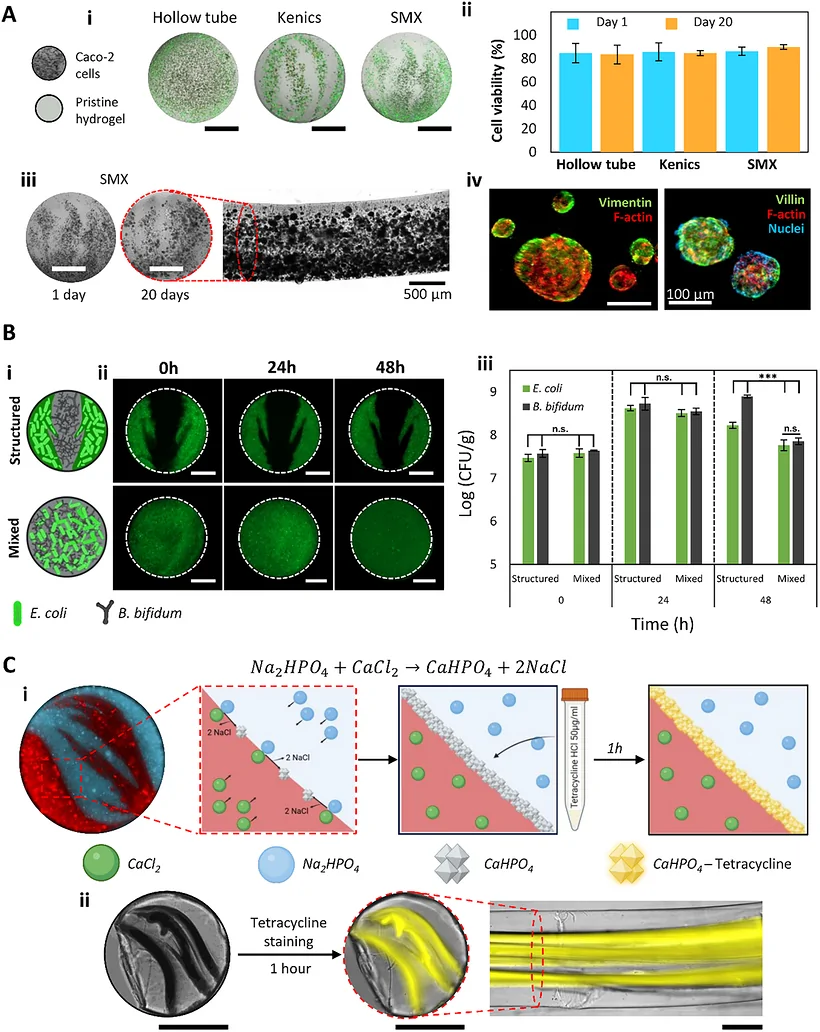
Structured soft materials fabricated by chaotic printing support cellular organization, microbial compartmentalization, and localized mineralization. (A) Caco‐2 cells were printed alongside a pristine hydrogel blend (2% alginate_LV_ + 3% GelMA) using three configurations: a hollow tube (no mixer), a 3‐element Kenics static mixer, and a 1‐element SMX static mixer. (i) Fluorescence micrographs of Live/Dead‐stained cross‐sections show the effect of internal architecture on cell distribution and viability. Scale bar: 500 µm. (ii) Quantification of cell viability across conditions, measured at day 1 and day 20 post‐printing, confirms high survival regardless of the printing modality. (iii) Brightfield images show cross‐sections at day 1 and day 20, along with a longitudinal view at day 20, from constructs printed with the 1‐element SMX mixer, highlighting the spontaneous formation of organized multicellular clusters. Scale bar: 500 µm. (iv) Immunofluorescence staining of day‐20 clusters shows expression of tissue‐relevant markers such as Vimentin and Villin (green), along with F‐actin (phalloidin, red) and nuclei (DAPI, blue). Scale bar: 100 µm. (B) Spatial organization and growth dynamics of *E. coli* (GFP‐expressing, green) and *Bifidobacterium bifidum (B. bifidum)* (non‐fluorescent) within bioprinted constructs using the 1‐element Mini‐SMX mixer. Structured constructs maintained spatially segregated bacterial populations, while controls with pre‐mixed inks displayed homogeneous distributions. (i) Schematic illustrations of cross‐sections depict the spatial arrangements for both conditions. (ii) Fluorescence micrographs of cross‐sections at 0, 24, and 48 h post‐printing show persistent compartmentalization in structured fibers versus full mixing in controls. Scale bar: 500 µm. (iii) Viable cell counts (Log CFU g^−1^) of *E. coli* and *B. bifidum* in both structured and mixed constructs over 48 h, based on triplicate samples (n  =  3). ^***^ *p* < 0.001; n.s., not significant. (C) Localized calcium phosphate precipitation within structured hydrogel fibers demonstrates spatially confined mineral domains enabled by chaotic printing, illustrating its potential for spatially programmed mineralization. Scale bar: 500 µm.

The hollow channel, which lacks internal elements and therefore does not promote stretching or folding of the inlet streams, does not generate significant new interface and is excluded from this comparative analysis. In contrast, the printheads containing static mixing elements rely on chaotic advection as the fundamental mechanism for microstructure generation. Although all static mixers considered here generate chaotic flow through successive reorientation and splitting of fluid streams under laminar conditions, each mixer produces unique flow characteristics and microarchitectural signatures associated to its geometry [29]. The Kenics configuration produces repeated helical twisting and splitting of the inlet streams, continuously reorienting and dividing the flow layers and thereby multiplying interfacial area. The Ross mixer induces discrete cross‐flow redirections that redistribute material layers and generate interfaces through localized shear and flow deflection. In contrast, the Mini‐SMX and SMX mixers—characterized by intersecting baffle structures—produce strong transverse flows and multi‐directional rearrangements of the streams, resulting in rapid proliferation of interfaces across the channel cross‐section.

Because these mixers generate chaotic advection, an exponential growth of interfacial area is expected in principle [29, 50, 51, 52]. This behavior is typically characterized by the Lyapunov exponent [53, 54], which quantifies the rate at which neighboring fluid elements separate in a chaotic flow. Consistent with this interpretation, Figure 3Dii, iii shows that the SMX geometry produces the highest rate of interface generation among the mixers evaluated, reflecting the strong stretching induced by its cross‐baffle topology and its correspondingly larger Lyapunov exponent [49, 55]. However, a key observation emerging from these results is that, within the short residence lengths relevant for chaotic printing, the interface growth is often better described by an approximately linear trend rather than by the exponential behavior expected in the asymptotic chaotic regime (Figure 3Dii). This occurs because only a limited number of reorientation‐splitting cycles take place within the small number of mixing elements used in printing applications, and during this initial period, the mixing process is still highly dependent on initial conditions and wall/geometry effects. The Kenics mixer represents a notable exception to this trend: even within this short mixing length, the interface generated by the Kenics mixer clearly follows an exponential growth pattern (as depicted by the yellow trend in Figure 3Dii). This behavior reflects the deterministic splitting and reorientation mechanism of the Kenics geometry, which produces the well‐known 2^*n*^lamellar multiplication is characteristic of this mixer.

These observations highlight an important conceptual distinction between classical mixing and chaotic printing. In conventional static mixer applications, the objective is complete homogenization, and therefore mixers with higher Lyapunov exponents—such as the SMX—are typically considered superior. In chaotic printing, however, the objective is not homogenization but rather the rapid generation of controlled microarchitecture within the printed filament. In this context, the relevant metric is not simply the asymptotic mixing efficiency but the rate of microarchitectural interface generation over short residence lengths, which depends not only on stretching efficiency but also on flow topology and inlet configuration.

Together, these results demonstrate that while all mixer geometries evaluated here can induce chaotic advection, their ability to generate microarchitecture within the limited residence lengths relevant for biofabrication varies significantly [29]. The SMX exhibits the highest instantaneous interface generation rates, while the Kenics geometry provides highly predictable and controlled lamellar structures. These differences illustrate the design‐dependent trade‐offs between interface generation rate, structural predictability, and operational energy requirements. For instance, the Mini‐SMX appears capable of generating high interface densities with a lower pressure penalty than the full SMX design, while the Kenics mixer achieves rapid interface multiplication with relatively moderate flow resistance.

Collectively, these results also indicate that the choice of mixer geometry for chaotic printing should not be based solely on classical mixing metrics but rather on the specific architectural objectives of the printing process, including desired feature size, structural regularity, and energetic efficiency.

Since different static mixers provide distinct balances between interface generation rate, pressure drop, shear exposure, and structural predictability, the choice of mixer should consider the intended application. For example, Kenics mixers provide highly predictable lamellar structures with moderate pressure losses and smoother shear profiles, making them particularly attractive for bioprinting applications involving shear‐sensitive cells. In contrast, SMX‐type mixers generate more aggressive interface proliferation and finer feature sizes, which may be advantageous for applications requiring very high interfacial area or complex material compartmentalization. Ross and Mini‐SMX geometries provide additional architectural motifs and intermediate hydrodynamic behaviors that further broaden the design space of chaotic printing.

Next, we provide a proof of concept of chaotic printing applications using different static mixers.

### 2.6 Structured Soft Materials Via Chaotic Printing Enable Spatial Control Across Biological and Chemical Systems

To explore the potential of chaotic printing to generate architected constructs with biological and chemical functionality, we applied this strategy across three distinct scenarios: mammalian cell culture, bacterial consortia, and localized mineralization.

Using different static mixer geometries, we fabricated hydrogel‐based constructs with controlled internal organization, providing proof‐of‐concept examples of how engineered microarchitectures can enable biologically and chemically relevant processes.

### 2.7 Spatially Guided Formation of Intestinal‐Like Microtissues

We first investigated whether chaotic printing could promote self‐organization of Caco‐1 cells during long‐term culture. Caco‐1 cells were printed within a pristine hydrogel blend (2% alginate_LV_ + 3% GelMA) using three configurations: a hollow tube (no mixer), a 3‐element Kenics static mixer, and a 1‐element SMX static mixer. Fluorescence micrographs of Live/Dead‐stained cross‐sections (Figure 4Ai) show high cell viability across all configurations. Quantification of viability at day 1 and day 20 confirmed that all conditions supported sustained cell survival (Figure 4Aii).

In Figure 4Aiii, we show a time‐course of constructs printed with the 1‐element SMX, including cross‐sections at days 1 and 20, and a longitudinal view at day 20, revealing the spontaneous emergence of organized multicellular clusters. Immunofluorescence staining of these clusters (Figure 4Aiv) revealed the expression of tissue‐relevant markers, including Villin and Vimentin (green), F‐actin (phalloidin, red), and DAPI‐stained nuclei (blue), suggesting that the printed microenvironment not only supports long‐term viability but also preserves functionality and enables tissue‐like organization.

This serves as simple proof‐of‐concept that the combination of continuous fabrication and chaotic microarchitectures can yield structurally complex, functional systems from a minimal setup. The microenvironment provided by chaotic printing was sufficiently permissive to support long‐term culture, self‐organization, and marker expression.

Importantly, this minimalistic system is inherently versatile: in previous work, we have shown that it can support the co‐extrusion of multiple materials, beyond two [23], including sacrificial phases to generate hollow channels resembling microvasculature [20], reinforcing components for enhanced mechanical performance [56], and phase‐specific materials to provide localized chemical and physical cues [22].

### 2.8 Compartmentalized Bacterial Consortia within Bioprinted Fibers

We then evaluated the potential of chaotic printing to spatially organize bacterial populations within hydrogel fibers. As a proof of concept, *Escherichia coli (E. coli)* (GFP‐expressing, facultative anaerobe) and *Bifidobacterium bifidum (B. bifidum)* (strict anaerobe) was bioprinted using the Mini‐SMX static mixer equipped with two inlets and a single mixing element. In structured constructs, the inks were loaded separately into each inlet, allowing the formation of a defined microarchitecture in which *E. coli* localized to the outer regions and *B. bifidum* to the core. This arrangement was designed to establish an oxygen gradient, where the peripheral *E. coli* could shield the inner *B. bifidum* from ambient oxygen, enhancing the survival and proliferation of the anaerobic species. Control constructs were printed with pre‐mixed bacteria, resulting in homogeneously distributed populations.

Importantly, both species showed comparable initial viability in structured and mixed constructs (>1 × 10^7^ CFU g^−1^ at 0 h), supporting valid comparisons between configurations. Schematic representations of both designs are shown in Figure 4Bi.

Fluorescence micrographs of cross‐sections taken at 0, 24, and 48 h (Figure 4Bii) revealed that structured constructs maintained spatial segregation of the bacterial populations, while mixed controls exhibited full homogenization. Quantification of viable cells over time (Figure 4Biii) showed that *B. bifidum* reached significantly higher counts in structured constructs (∼1 × 10^9^ CFU g^−1^ at 48 h) compared to mixed ones, consistent with the hypothesized protective effect of spatial compartmentalization. This result supports the notion that the rational design of the construct—dictating microstructure during printing—can generate microenvironments favorable to oxygen‐sensitive species.

More broadly, these findings demonstrate that chaotic printing enables the creation of heterogeneous microbial landscapes with tailored spatial arrangements. While in this example we engineered an oxygen‐limited niche, the same approach could be adapted to control gradients of nutrients, metabolites (e.g., for cross‐feeding), biomolecules, or mechanical properties. Such tunable compartmentalization holds promise not only for anaerobe cultivation but as a versatile platform to fabricate structured co‐cultures for gut‐mimetic systems and beyond.

### 2.9 Localized Biomineralization within Hydrogel Fibers

We explored whether chaotic printing could spatially guide site‐specific chemical processes within soft hydrogel constructs by inducing localized biomineralization. As a proof of concept, phosphate‐rich and calcium‐rich gelatin inks were co‐extruded using a Ross static mixer with three elements, generating interdigitated domains with distinct chemical compositions (Figure 4C). The controlled spatial segregation of these domains enabled calcium phosphate precipitation at defined internal interfaces within the printed fibers (Figure 4Ci). Localized mineral domains were visualized using tetracycline‐based fluorescence staining (Figure 4Cii). Of note, both inks share the same continuous gelatin‐based matrix and differ primarily in their ionic composition (phosphate‐rich versus calcium‐rich). Therefore, despite the existence of chemically distinct domains, the construct remains structurally continuous at the polymeric level. Furthermore, enzymatic crosslinking mediated by transglutaminase occurs throughout the entire filament after extrusion, generating a mechanically integrated hydrogel network that spans across the different microregions and interfaces.

Beyond biomineralization, this strategy illustrates how chaotic printing can be used to spatially program localized chemical transformations within soft materials, opening opportunities to engineer composite hydrogels with functional gradients or hybrid biofabricated systems.

Indeed, one attractive aspect of chaotic printing is precisely the possibility of engineering controlled internal architectures while maintaining continuity within the bulk material. This capability could enable the fabrication of multifunctional composite hydrogels and bioinspired soft materials with spatially programmed mechanical, chemical, or biological properties.

The examples shown in Figure 4 are intended to demonstrate the potential of chaotic printing to organize biological and chemical systems in ways that may ultimately enable more advanced, functional engineered materials and tissues.

## 3 Conclusions

In this study, we established chaotic printing as a versatile platform for engineering soft hydrogel constructs with finely structured internal architectures. By leveraging static mixers, namely the Kenics, Ross, mini‐SMX, and SMX static mixers, we generated deterministic lamellar patterns using different hydrogel systems and crosslinking mechanisms—including ionic (alginate), photoinitiated (GelMA), and enzymatic (gelatin with transglutaminase) stabilization. These proofs of concept highlight the method's adaptability and its potential to be extended to a broader range of cross‐linkable or solidifiable polymeric materials.

To better understand the internal flow behavior across the different static mixer geometries, we conducted computational fluid dynamics (CFD) simulations using representative models of each mixer. This CFD‐based analysis demonstrates that the selection of the static mixer geometry for chaotic printheads has implications that extend beyond purely microarchitectural considerations. Mixer geometry also governs the magnitude and distribution of shear stresses and pressure drops within the printhead, parameters that are particularly relevant for bioprinting applications where cells may be sensitive to elongational stresses arising from strong velocity gradients. Our results indicate that the Kenics static mixer offers a particularly advantageous compromise between rapid interface generation, controlled shear levels, and moderate pressure losses, making it a robust geometry for chaotic printing applications involving shear‐sensitive living materials. Importantly, although the Kenics mixer provides this balanced performance, the Ross and SMX‐type mixers remain viable alternatives with distinct hydrodynamic signatures. The availability of multiple mixer geometries, therefore, broadens the engineering design space of chaotic printing and enables adaptation to diverse materials, processing conditions, and architectural and/or biological requirements.

We also demonstrated that the microstructures generated by different chaotic printhead configurations enabled precise spatial control over mammalian and microbial populations, preserving compartmentalization and supporting long‐term viability. Furthermore, we demonstrated the system's capacity to guide localized chemical reactions within soft constructs, exemplified by the formation of calcium phosphate precipitates along internal interfaces. This suggests the potential of chaotic printing to fabricate constructs with functional gradients or hybrid living–nonliving features, and to explore interfacial reactivity as a tool for material synthesis or modulation.

Unlike many fabrication strategies that generate internal architecture through sequential deposition, externally applied fields, or post‐processing assembly, chaotic printing creates complex microarchitectures through passive flow transformations occurring within the printhead itself. This enables the continuous generation of finely structured, multimaterial constructs in a single fabrication step. Importantly, multiple materials, cells, microorganisms, particles, or reactive species can be spatially organized within the same filament while maintaining overall structural continuity. Beyond demonstrating this capability, the present work establishes static mixer geometry as a design parameter for controlling microarchitecture, interface generation, shear exposure, and pressure losses. By extending chaotic printing beyond traditional Kenics‐based systems to include Ross, Mini‐SMX, and SMX geometries, we expand the architectural design space available to researchers. Together, these features distinguish chaotic printing as a simple, scalable, and biologically compatible strategy for encoding internal organization in soft and living materials.

Looking ahead, chaotic printing could facilitate the development of next‐generation engineered systems—from structured microbial co‐cultures and tissue interfaces to soft composites with spatially encoded properties. Future directions may include exploring sensing capabilities that emerge from interfacial reactivity, as well as using this architecture to direct nutrient diffusion, metabolite exchange, or even the synthesis of inorganic phases within hydrogel scaffolds. With its simplicity, scalability, and architectural precision, chaotic printing offers a powerful foundation for structuring complexity in soft matter systems.

## 4 Materials and Methods

Low viscosity alginate (alginate_LV_), high viscosity alginate (alginate_HV_)calcium chloride, tween 80, and minimum essential medium (EMEM) were purchased from Sigma‐Aldrich (St. Louis, USA). Lithium phenyl‐2,4,6‐trimethylbenzoylphosphinate (LAP), acquired from Allevi (Philadelphia, USA), was used as photo‐initiator for GelMA‐containing inks. MDA‐MB 231 cells (HTB‐26, ATCC) or Caco‐2 cells (HTB‐37, ATCC) were purchased from ATCC (USA). Live/Dead Cell imaging kit was purchased from Invitrogen (Carlsbad, USA).

### 4.1 Design of Chaotic Printheads

Cylindrical printheads containing each type of static mixer (i.e., SMX, modified SMX, Ross, Kenics, or Ross–Kenics mixer) were designed using SolidWorks. Miniaturized versions of each static mixer were established according to previous reports. Printheads were fabricated by stereolithography using a FormLabs 3 printer (FormLabs, Somerville, USA) and clear resin (FLGPCL04, FormLabs). Nozzle tips with an outlet diameter of 1, 0.5, or 0.2 mm were coupled to the printheads for tuning the diameter of filaments or spheres. Detailed information about the geometry and size of the chaotic printheads is presented in the Results and Discussion section (Figure 2).

### 4.2 Preparation of Fluorescent Inks

*Escherichia coli* expressing either red or green fluorescent protein were as microparticles used to visualize the spatial distribution generated by chaotic printheads. Bacteria were grown on Luria–Bertani (LB) medium (Sigma–Aldrich, USA) with 1 µL/mL of chloramphenicol, and incubated at 37°C and 120 rpm for 24 h. Then, bacteria‐containing culture media were centrifuged at 8000 rpm for 10 min, and the bacterial pellet was mixed with a blend of 3% GelMA and 2% alginate_LV_.

### 4.3 Chaotic Printing Setup

Three chaotic printing systems were used throughout the manuscript: wet‐printing, dripping, and direct ink writing. For wet‐printing, 2% alginate‐based inks containing green or red fluorescent bacteria were co‐extruded through a chaotic printhead using a commercial syringe pump Fusion 200 (Chemyx, Stafford, USA) at a flow rate of 1.5 mL min^−1^. The nozzle outlet was immersed in a 2% calcium chloride solution to crosslink alginate molecules and obtain a solid filament.

In dripping experiments, a similar setup was used with slight modifications. Tween 80 at 1 g L^−1^ was added to the 2% calcium chloride bath to decrease the surface tension [59]. Chaotic printheads were placed 15 cm above the calcium chloride solution, and the co‐extruded inks (2% alginate_LV_) were dripped to obtain spheres.

For direct‐ink writing, chaotic printheads were connected to a 22G nozzle and filled with cooled 30% pluronic F‐127 containing fluorescent microparticles. Pre‐filled printheads were placed in a commercial 3D‐bioprinter (BioX, Cellink, California, USA). Printhead inlets were connected to the pressure line of the BioX using a plastic bifurcation. Fluorescent inks were deposited on Petri dishes at a linear velocity of 5–8 mm s^−1^ and a pressure of 120–130 kPa. Pre‐ and post‐flow modes were activated.

### 4.4 Bioprinting Experiments

Caco‐2 cells were cultured in EMEM medium supplemented with 10% fetal bovine serum (FBS), respectively. Upon reaching confluency, cells were detached and mixed with a blend of 3% GelMA and 2% alginate_LV_ at a concentration of 3 x 10^6^ cells mL^−1^. The as‐prepared bioink was co‐extruded with the same ink composition through a chaotic printhead, and cross‐linked by a two‐step process: aqueous solution of 2% CaCl_2_ for 1 min, followed by UV light at 365 nm (100 mW cm^−2^) for 30 s [21]. Cell‐laden constructs were cultivated in EMEM medium supplemented with 10% FBS. Caco‐2 cells were used at passages 5 to 15.

### 4.5 Assessment of Cell Viability

Post‐printing viability of cells was determined by the Live/Dead assay. Samples were incubated for 30 min at 37°C in the presence of calcein AM and ethidium homodimer (EtHD1). Then, they were washed with PBS and imaged using an Axio Observer.Z1 microscope (Zeiss, Germany) equipped with Colibri.2 LED illumination and an Apotome.2 system (Zeiss). Imaging and image analysis were performed as detailed in our previous report [21].

### 4.6 Bacteria Printing

*E. coli* (EcGFP) was cultured at 37°C with shaking at 150 rpm in Luria–Bertani (LB) broth Lennox medium (Sigma–Aldrich), supplemented with 1 µL mL^−^^1^ chloramphenicol to maintain the recombinant plasmid. *B. bifidum* was grown in de Man, Rogosa, and Sharpe (MRS) medium (Merck, Darmstadt, Germany) at 37°C. Both strains were incubated for 24 h under their respective growth conditions. Subsequently, 5 mL of each bacterial culture was centrifuged at 7000 × g for 10 min at 4°C, and the supernatants were discarded. For bioprinting experiments, the optical densities (OD_600_) of the bacterial bioinks were adjusted to 0.3 and 0.1 for EcGFP and *B. bifidum*, respectively, to enable balanced growth and ensure experimental reproducibility, based on prior experience with cocultures in bacterial chaotic bioprinting [36, 57, 58]. The resulting pellets were resuspended directly in a 2% sterile alginate_HV_ solution (Sigma–Aldrich).

For microstructured coculture, each bacterial bioink was deposited in separate 3 mL sterile syringes and connected to a previously sterilized mini‐SMX printhead equipped with two top‐positioned inlets and one mixing element. For well‐mixed co‐culture printing, the bioinks were pre‐mixed, combining both in the same tube, and this mixture was then used to load the two syringes and perform the printing.

In all experiments, the bioinks were coextruded at a flow rate of 1.5 mL min^−^^1^ at room temperature, while the printhead outlet was immersed in a 2% CaCl_2_ solution for crosslinking. Bacteria‐laden constructs were cultured in 50 mL conical tubes containing 7 mL of brain heart infusion (BHI) medium (Becton, Dickinson, and Company). All bioprinting experiments were performed aseptically inside a laminar flow cabinet, and the bacteria‐laden fibers were cultivated at 37°C with shaking at 50 rpm during 48 h.

### 4.7 Bacterial Cell Viability

The colony forming unit (CFU) and spread plate counting assays were performed to determine the viability of the samples. The number of CFUs was assessed by disaggregating and homogenizing 0.006 g of the printed filament in a mixture of BHI medium and sodium citrate solution (1:1). The homogenized sample was sequentially diluted in PBS by tenfold serial dilutions. Sequential dilutions were seeded at least in duplicate on Petri dishes containing LB agar (Sigma–Aldrich), and MRS agar (Merck, Darmstadt, Germany). The LB agar was incubated under aerobic conditions at 37°C, while the MRS agar remained under an anaerobic atmosphere at 37°C for at least 2 days. The collected data were multiplied by the dilution and proportion factors, and the results in CFU g^−^^1^ were plotted on a logarithmic scale.

### 4.8 Mineralization Experiments

For mineralization experiments, gelatin‐based inks with distinct ionic compositions were prepared. A phosphate‐rich ink was obtained by dissolving gelatin powder (Sigma–Aldrich, G1890) in a 0.25 m sodium hydrogen phosphate (Na_2_HPO_4_; Sigma–Aldrich, S3264) solution under continuous agitation until complete dissolution. In parallel, a calcium‐rich ink was prepared by dissolving gelatin powder (Sigma–Aldrich, G1890) in a 0.25 m calcium chloride (CaCl_2_; Sigma–Aldrich, C1016) solution.

To enable enzymatic crosslinking, both pre‐inks were mixed with a transglutaminase (TGase) stock solution prepared by dissolving TGase powder (Modernist Pantry, 1203) in distilled water at 15% (w/v), followed by sterile filtration using a 0.22 µm syringe filter. The final ink composition consisted of 10% (w/v) gelatin and 1.5% (w/v) TGase. Phosphate‐ and calcium‐rich inks were loaded into separate syringes and co‐extruded through a printhead equipped with three Ross static mixer elements at a flow rate of 0.5 mL min^−^^1^ per inlet. Printing was performed with the nozzle immersed in distilled water maintained at 4°C.

Following printing, the resulting filaments were cut into approximately 2 cm segments and fixed in 4% paraformaldehyde (PFA) for 15 min at room temperature. Samples were washed three times with distilled water and subsequently incubated in a tetracycline hydrochloride solution (50 µg mL^−^^1^; Goldbio, T‐101) for 1 h at room temperature, protected from light. Tetracycline staining was used to visualize calcium phosphate deposition within the printed constructs. Fluorescence imaging was performed using an Axio Observer.Z1 microscope (Zeiss, Germany) with an LED illumination intensity of 20% and an exposure time of 150 ms in the CNF channel.

### 4.9 Computational Simulations

Computational fluid dynamics simulations were performed using ANSYS Fluent 2020 Software. The co‐extrusion of fluids through each chaotic printhead was mimicked using a multi‐phase model as described in our previous report [21].

### 4.10 Microscopy Analyses

Printed filaments or spheres were placed in Petri dishes containing deionized water, or PBS when mammalian cells were embedded in the construct, to prevent dehydration during imaging. The microarchitecture of printed objects was observed using an Axio Observer.Z1 microscope (Zeiss, Germany) equipped with Colibri.2 LED illumination and an Apotome.2 system (Zeiss). Fluorescence micrographs were acquired in wide‐field or Z‐stack modes using appropriate filter sets for each fluorophore. Imaging parameters were adjusted depending on the experimental condition and fluorophore, as specified in the corresponding figure captions or experimental subsections.

### 4.11 Statistical Analyses

Data were analyzed by one‐way ANOVA using Minitab 17. Differences between means were considered as significant at a *p*‐value ≤ 0.05.

## Author Contributions

**Carlos Fernando Ceballos‐González**: investigation, methodology, validation, Writing – review and editing, Writing – original draft, formal analysis. **Irving Isaí Rendón‐Moreno**: investigation, validation. **Grissel Trujillo‐de Santiago**: conceptualization, funding acquisition, supervision, project administration. **Héctor Ambriz‐González**: investigation, methodology, formal analysis. **Edna Johana Bolívar‐Monsalve**: investigation, methodology, writing – review and editing, writing – original draft, formal analysis. **Mario Moisés Alvarez**: conceptualization, funding acquisition, writing – original draft, supervision. **Ariel Cantoral‐Sánchez**: investigation, validation, methodology, data curation. **Diego Alonso Quevedo‐Moreno**: validation, visualization, software, formal analysis, methodology, data curation.

## Conflicts of Interest

The authors declare no conflicts of interest.

## Data Availability

The data that support the findings of this study are available from the corresponding author upon reasonable request.

## References

[1] S. Ferrachat and Y. Ricard , “Regular vs. chaotic Mantle Mixing,” Earth and Planetary Science Letters 155, no. 1 (1998): 75–86, 10.1016/S0012-821X(97)00200-8.

[2] A. Kurakin , “The Self‐organizing Fractal Theory as a Universal Discovery Method: The Phenomenon of Life,” Theoretical Biology and Medical Modelling 8, no. 1 (2011): 4, 10.1186/1742-4682-8-4. PMC3080324 21447162

[3] Y. Beygelzimer , R. Kulagin , P. Fratzl , and Y. Estrin , “The Earth's Lithosphere Inspires Materials Design,” Advanced Materials 33, no. 3 (2021): 2005473. 10.1002/adma.202005473 PMC11468334 33300235

[4] A. Velasco‐Hogan , J. Xu , and M. A. Meyers , “Additive Manufacturing as a Method to Design and Optimize Bioinspired Structures,” Advanced Materials 30, no. 52 (2018): 1800940, 10.1002/adma.201800940. 30133816

[5] A. S. Perera and M.‐O. Coppens , “Re‐designing Materials for Biomedical Applications: From Biomimicry to Nature‐Inspired Chemical Engineering,” Philosophical Transactions of the Royal Society A: Mathematical, Physical and Engineering Sciences 377, no. 2138 (2019): 20180268, 10.1098/rsta.2018.0268. PMC6335285 30967073

[6] J. Wei , F. Pan , H. Ping , et al., “Bioinspired Additive Manufacturing of Hierarchical Materials: From Biostructures To Functions,” Research 6 (2023): 0164. 10.34133/research.0164 PMC10254471 37303599

[7] K. Cremin , S. J. N. Duxbury , J. Rosko , and O. S. Soyer , “Formation and Emergent Dynamics of Spatially Organized Microbial Systems,” Interface Focus 13, no. 2 (2023): 20220062, 10.1098/rsfs.2022.0062. PMC9912014 36789239

[8] S. Tsitkov and H. Hess , “Design Principles for a Compartmentalized Enzyme Cascade Reaction,” ACS Catalysis 9, no. 3 (2019): 2432–2439, 10.1021/acscatal.8b04419.

[9] Y. Zhang and H. Hess , “Toward Rational Design of High‐efficiency Enzyme Cascades,” ACS Catalysis 7, no. 9 (2017): 6018–6027, 10.1021/acscatal.7b01766.

[10] K. Ruan , Y. Tian , Y. Tian , M. Li , K. Zhou , and J. Gu , “Architecting Optimized Thermal Conduction Pathways in Colonnade‐structured Polydimethylsiloxane‐based Thermal Interface Materials by Direct Ink Writing,” Science Bulletin 71, no. 8 (2026): 2033–2043, 10.1016/j.scib.2026.03.011. 41864785

[11] Y. Guo , S. Wang , H. Zhang , et al., “Consistent Thermal Conductivities of Spring‐Like Structured Polydimethylsiloxane Composites Under Large Deformation,” Advanced Materials 36, no. 39 (2024): 2404648, 10.1002/adma.202404648. 38970529

[12] Y. Zhang , K. Ruan , K. Zhou , and J. Gu , “Controlled Distributed Ti_3_C_2_T_x_ Hollow Microspheres on Thermally Conductive Polyimide Composite Films for Excellent Electromagnetic Interference Shielding,” Advanced Materials 35, no. 16 (2023): 2211642, 10.1002/adma.202211642. 36703618

[13] S. O'Halloran , A. Pandit , A. Heise , and A. Kellett , “Two‐photon Polymerization: Fundamentals, Materials, and Chemical Modification Strategies,” Advancement of Science 10, no. 7 (2023): 2204072. 10.1002/advs.202204072 PMC9982557 36585380

[14] J. Yang , Q. He , L. Liu , et al., “Anti‐scattering Light Focusing by Fast Wavefront Shaping Based on Multi‐pixel Encoded Digital‐micromirror Device,” Light: Science Applied 10, no. 1 (2021): 149, 10.1038/s41377-021-00591-w. PMC8292544 34285183

[15] Y. Geng , S. Ling , J. Huang , and J. Xu , “Multiphase Microfluidics: Fundamentals, Fabrication, and Functions,” Small 16, no. 6 (2020): 1906357, 10.1002/smll.201906357. 31913575

[16] G. Zhang , W. Li , M. Yu , et al., “Electric‐Field‐Driven Printed 3D Highly Ordered Microstructure With Cell Feature Size Promotes the Maturation of Engineered Cardiac Tissues,” Advancement of Science 10, no. 11 (2023): 2206264, 10.1002/advs.202206264. PMC10104649 36782337

[17] G. Trujillo‐de Santiago and M. M. Alvarez , “Together but Not Scrambled: A Perspective on Chaotic Printing/Bioprinting,” Aggregate 5, no. 4 (2024): 548, 10.1002/agt2.548.

[18] G. Trujillo‐de Santiago , M. M. Alvarez , M. Samandari , et al., “Chaotic Printing: Using Chaos to Fabricate Densely Packed Micro‐ and Nanostructures at High Resolution and Speed,” Materials Horizons 5, no. 5 (2018): 813–822, 10.1039/C8MH00344K. PMC11309736 39119486

[19] C. Chávez‐Madero , M. D. De León‐Derby , M. Samandari , et al., “Using Chaotic Advection for Facile High‐throughput Fabrication of Ordered Multilayer Micro‐and Nanostructures: Continuous Chaotic Printing,” Biofabrication 12, no. 3 (2020): 035023, 10.1088/1758-5090/ab84cc. 32224513

[20] E. J. Bolívar‐Monsalve , C. F. Ceballos‐González , C. Chavez‐Madero , et al., “One‐Step Bioprinting of Multi‐Channel Hydrogel Filaments Using Chaotic Advection: Fabrication of Pre‐Vascularized Muscle‐Like Tissues,” Advanced Healthcare Materials 11, no. 24 (2022): 2200448, 10.1002/adhm.202200448. 35930168

[21] E. J. Bolívar‐Monsalve , C. F. Ceballos‐González , K. I. Borrayo‐Montaño , et al., “Continuous Chaotic Bioprinting of Skeletal Muscle‐Like Constructs,” Bioprinting 21 (2021): 00125.

[22] S. C. Pedroza‐González , M. P. Pérez González , A. F. Ochoa Tiscareño , et al., “Synergistic Physical and Chemical Cues Enhance Cellularization in Compartmentalized Microchannel Fibers Supplemented With Mesoporous Bioactive Glass Nanoparticles,” ACS Materials Letters 7, no. 8 (2025): 2765–2775, 10.1021/acsmaterialslett.5c00250.

[23] C. F. Ceballos‐González , E. J. Bolívar‐Monsalve , D. A. Quevedo‐Moreno , et al., “Plug‐and‐Play Multimaterial Chaotic Printing/Bioprinting to Produce Radial and Axial Micropatterns in Hydrogel Filaments (Adv. Mater. Technol. 17/2023),” Advanced Materials Technology 8, no. 17 (2023): 2370083, 10.1002/admt.202370083.

[24] C. F. Ceballos‐González , E. J. Bolívar‐Monsalve , S. Velásquez‐Marín , et al., “Chaos‐Assisted Production of Micro‐Architected Spheres (CAPAS),” Small 21 (2024): 2402221, 10.1002/smll.202402221. 39161204

[25] M. Samandari , F. Alipanah , K. Majidzadeh‐A , M. M. Alvarez , G. Trujillo‐de Santiago , and A. Tamayol , “Controlling Cellular Organization In Bioprinting Through Designed 3D Microcompartmentalization,” Applied Physics Reviews 8, no. 2 (2021): 021404. 10.1063/5.0040732 PMC8100992 34084254

[26] M. Pezo , L. Pezo , A. Jovanović , B. Lončar , and R. Čolović , “DEM/CFD Approach for Modeling Granular Flow in the Revolving Static Mixer,” Chemical Engineering Research and Design 109 (2016): 317–326, 10.1016/j.cherd.2016.02.003.

[27] A. Jovanović , M. Pezo , L. Pezo , and L. Lević , “DEM/CFD Analysis of Granular Flow in Static Mixers,” Powder Technology 266 (2014): 240–248, 10.1016/j.powtec.2014.06.032.

[28] M. K. Singh , P. D. Anderson , and H. E. H. Meijer , “Understanding and Optimizing the SMX Static Mixer,” Macromolecular Rapid Communications 30, no. 4 (2009): 362–376, 10.1002/marc.200800710. 21706612

[29] H. E. H. Meijer , M. K. Singh , and P. D. Anderson , “On the Performance of Static Mixers: A Quantitative Comparison,” Progress in Polymer Science 37, no. 10 (2012): 1333–1349, 10.1016/j.progpolymsci.2011.12.004.

[30] U. G. K. Wegst , H. Bai , E. Saiz , A. P. Tomsia , and R. O. Ritchie , “Bioinspired Structural Materials,” Nature Materials 14, no. 1 (2015): 23–36. 10.1038/nmat4089 25344782

[31] P. Fratzl and R. Weinkamer , “Nature's Hierarchical Materials,” Progress in Materials Science 52, no. 8 (2007): 1263–1334, 10.1016/j.pmatsci.2007.06.001.

[32] R. Lakes , “Materials With Structural Hierarchy,” Nature 361, no. 6412 (1993): 511–515, 10.1038/361511a0.

[33] R. C. Thayer , F. I. Allen , and N. H. Patel , “Structural Color in Junonia Butterflies Evolves by Tuning Scale Lamina Thickness,” Elife 9 (2020): 52187. 10.7554/eLife.52187 PMC7138606 32254023

[34] V. J. Lloyd , S. L. Burg , J. Harizanova , et al., “The Actin Cytoskeleton Plays Multiple Roles in Structural Colour Formation in Butterfly Wing Scales,” Nature Communications 15, no. 1 (2024): 4073, 10.1038/s41467-024-48060-3. PMC11106069 38769302

[35] G. D. Bixler and B. Bhushan , “Bioinspired Rice Leaf and Butterfly Wing Surface Structures Combining Shark Skin and lotus Effects,” Soft Matter 8, no. 44 (2012): 11271, 10.1039/c2sm26655e.

[36] C. F. Ceballos‐González , E. J. Bolívar‐Monsalve , D. A. Quevedo‐Moreno , et al., “High‐throughput and Continuous Chaotic Bioprinting of Spatially Controlled Bacterial Microcosms,” ACS Biomaterials Science Engineering 7, no. 6 (2021): 2408–2419, 10.1021/acsbiomaterials.0c01646. 33979127

[37] D. M. Hobbs and F. J. Muzzio , “The Kenics Static Mixer: A Three‐dimensional Chaotic Flow,” Chemical Engineering Journal 67, no. 3 (1997): 153–166, 10.1016/S1385-8947(97)00013-2.

[38] S. V. Murphy and A. Atala , “3D Bioprinting of Tissues and Organs,” Nature Biotechnology 32, no. 8 (2014): 773–785, 10.1038/nbt.2958. 25093879

[39] I. T. Ozbolat and M. Hospodiuk , “Current Advances and Future Perspectives in Extrusion‐based Bioprinting,” Biomaterials 76 (2016): 321–343, 10.1016/j.biomaterials.2015.10.076. 26561931

[40] M. Korpyś , M. Iwaniszyn , K. Sindera , M. Suwak , A. Gancarczyk , and A. Kołodziej , “Flow Phenomena in Laminar Flow Through Streamlined and Sharp‐edged Short Monolithic Structures,” Scientific Reports 13, no. 1 (2023): 15742. 10.1038/s41598-023-42568-2 PMC10514183 37735537

[41] K. Urbanowicz , A. Bergant , M. Stosiak , A. Deptuła , and M. Karpenko , “Navier‐Stokes Solutions for Accelerating Pipe Flow—A Review of Analytical Models,” Energies 16, no. 3 (2023): 1407, 10.3390/en16031407.

[42] D. M. Hobbs and F. J. Muzzio , “Reynolds Number Effects on Laminar Mixing in the Kenics Static Mixer,” Chemical Engineering Journal 70, no. 2 (1998): 93–104, 10.1016/S0923-0467(98)00065-7.

[43] D. M. Hobbs , P. D. Swanson , and F. J. Muzzio , “Numerical Characterization of Low Reynolds Number Flow in the Kenics Static Mixer,” Chemistry Engineering Science 53, no. 8 (1998): 1565–1584, 10.1016/S0009-2509(97)00132-2.

[44] M. K. Singh , T. G. Kang , P. D. Anderson , H. E. H. Meijer , and A. N. Hrymak , “Analysis and Optimization of Low‐Pressure Drop Static Mixers,” Aiche Journal 55, no. 9 (2009): 2208–2216, 10.1002/aic.11846.

[45] J. M. Zalc , E. S. Szalai , F. J. Muzzio , and S. Jaffer , “Characterization of Flow and Mixing in an SMX Static Mixer,” Aiche Journal 48, no. 3 (2002): 427–436, 10.1002/aic.690480303.

[46] N. Gayen , S. Swamy , S. Hari , and S. H. Sonawane , “Laminar mixing of Newtonian and non‐Newtonian fluids in SMX static mixer,” Canadian Journal of Chemical Engineering 102, no. 8 (2024): 2768–2785, 10.1002/cjce.25232.

[47] P. J. McCauley , C. A. Fromen , and A. V. Bayles , “Cell Viability in Extrusion Bioprinting: The Impact of Process Parameters, Bioink Rheology, and Cell Mechanics,” Rheologica Acta 64, no. 9 (2025): 497–515, 10.1007/s00397-025-01504-z.

[48] H.‐Q. Xu , J.‐C. Liu , Z.‐Y. Zhang , and C.‐X. Xu , “A Review on Cell Damage, Viability, and Functionality During 3d Bioprinting,” Military Medical Research 9, no. 1 (2022): 70. 10.1186/s40779-022-00429-5 PMC9756521 36522661

[49] D. Rauline , J.‐M. Le Blévec , J. Bousquet , and P. A. Tanguy , “A Comparative Assessment of the Performance of the Kenics and SMX Static Mixers,” Chemical Engineering Research and Design 78, no. 3 (2000): 389–396, 10.1205/026387600527284.

[50] L. von Damnitz and D. Anders , “A Review on the Mixing Quality of Static Mixers,” ChemEngineering 9, no. 6 (2025): 128.

[51] F. Jiang , K. S. Drese , S. Hardt , M. Küpper , and F. Schönfeld , “Helical Flows and Chaotic Mixing in Curved Micro Channels,” Aiche Journal 50, no. 9 (2004): 2297–2305, 10.1002/aic.10188.

[52] D. V. Khakhar , H. Rising , and J. M. Ottino , “Analysis of Chaotic Mixing in Two Model Systems,” Journal of Fluid Mechanics 172 (1986): 419–451, 10.1017/S0022112086001805.

[53] J. M. Ottino , “Mixing, Chaotic Advection, and Turbulence,” Annual Review of Fluid Mechanics 22, no. 1 (1990): 207–254, 10.1146/annurev.fl.22.010190.001231.

[54] M. A. Ghorbani , O. Kisi , and M. Aalinezhad , “A Probe into the Chaotic Nature of Daily Streamflow Time Series by Correlation Dimension and Largest Lyapunov Methods,” Applied Mathematical Modelling 34, no. 12 (2010): 4050–4057, 10.1016/j.apm.2010.03.036.

[55] P. A. Tanguy , L. Fradette , M. Heniche , and S. A. Jaffer , CFD Simulations of Static Mixers: A Survey, in Mixing and Compounding of Polymers ( Co. KG, 2009), 299–335, 10.3139/9783446433717.

[56] A. Cavero‐Arrivasplata , D. H. Hernández‐Medina , I. I. Rendón‐Moreno , et al., “Multimaterial Chaotic Printing of Reinforced and Prevascularized Hydrogel filaments: Fabrication of Mechanical Robust Constructs for Long‐term Muscle Tissue Culture,” Biomaterials Science 13, no. 23 (2025): 6598–6612, 10.1039/D4BM01674B. 40787729

[57] A. Cantoral‐Sánchez , O. E. Solís‐Pérez , F. J. Flores‐Loera , et al., “Biofabrication of Microstructured Bacterial Ecosystems Using Chaotic Bioprinting: Advancing in Vitro Research for Microbial Engineering,” Biofabrication 17, no. 3 (2025): 035015, 10.1088/1758-5090/add568. 40334674

[58] F. J. Flores‐Loera , A. Cantoral‐Sánchez , L. F. Carmona‐Ramirez , et al., “Bioprinting microbial harmony: Engineering spatially organized probiotic ecosystems via chaotic bioprinting,” Bioprinting 52 (2025): e00453, 10.1016/j.bprint.2025.e00453.

[59] E.‐S. Chan , B.‐B. Lee , P. Ravindra , and D. Poncelet , “Prediction Models for Shape and Size of ca‐alginate Macrobeads Produced Through Extrusion–Dripping Method,” Journal of Colloid & Interface Science 338, no. 1 (2009): 63–72, 10.1016/j.jcis.2009.05.027. 19604515

